# Imaging findings in a case of stand up paddle surfer’s myelopathy

**DOI:** 10.1259/bjrcr.20150004

**Published:** 2015-03-18

**Authors:** M E Klontzas, A Hatzidakis, A H Karantanas

**Affiliations:** Department of Medical Imaging, University Hospital of Heraklion, University of Crete, Heraklion-Crete, Greece

## Abstract

Stand up paddle (SUP) surfing, a variant of ocean surfing, is becoming very popular because it can be performed at any level of difficulty and thus attracts athletes from a wide range of ages. Unlike ocean surfing, limited data exist on injuries related to SUP surfing. We report the first case of a 28-year-old athlete who developed myelopathy during his first SUP surfing session. Clinical examination revealed severe neurological deficit, which had not subsided fully at the 28-month follow-up.

Stand up paddle (SUP) surfing is a rapidly evolving type of surfing where surfers lie prone on a surfboard with their spine hyperextended and use their bare hands to propel away from the coast. As soon as they reach sufficient wave heights, they use a paddle to propel themselves across the sea surface. No traumatic or non-traumatic complications have been reported to date by such surfers, and we hereby present the first case of SUP surfer’s myelopathy.

## CLINICAL PRESENTATION

A 28-year-old fit male (194 cm, 87 kg), residing on the coast in the eastern mainland of Greece, attempted his first SUP surfing session without any previous experience. The daily weather forecast reported a north–east wind direction, wind speed of 6 Bft and possible wave height of 80 cm. The athlete used an all-round surfboard (volume 180 l, length 12 inches, width 36 inches), which is suitable for beginners. He undertook his session between 12:00 and 16:00, which is the warmest time of the day with temperatures reaching 38 ^o^C on average. After a 4-h non-stop session, no injuries or trauma were reported. During this 4-h practice, he recalled one short break for hydration. 60min after ending surfing, he began to feel a pain in his back that rapidly progressed to 10/10 on a pain scale of 1–10. 2h later, the pain remained intense and he developed paraesthesia in his bilateral lower limbs.

## DIFFERENTIAL DIAGNOSIS

The patient’s medical history was free of any diseases. There was no recent history of any kind of viral disease, for example, influenza. Professionally, he taught football in high school academies. He undertook regular physical training himself, including basketball three times a week. In the emergency department, the clinical examination of respiratory and cardiovascular systems, mental status and cranial nerves was within normal limits. No signs of heat-related illness were discovered. The patient was unable to stand or walk, and the neurological examination showed reduced light-touch and pinprick sensation throughout both lower extremities.

## INVESTIGATIONS/IMAGING FINDINGS

Following hospitalization in a university hospital 4 h after the onset of symptoms, there was complete loss of sensation in the lower part of the body, paraparesis and loss of bladder and bowel function. Urodynamic evaluation disclosed a neurogenic bladder with no flow. The complete laboratory work-up, including analysis of the cerebrospinal fluid, was normal.

**Figure 1.**
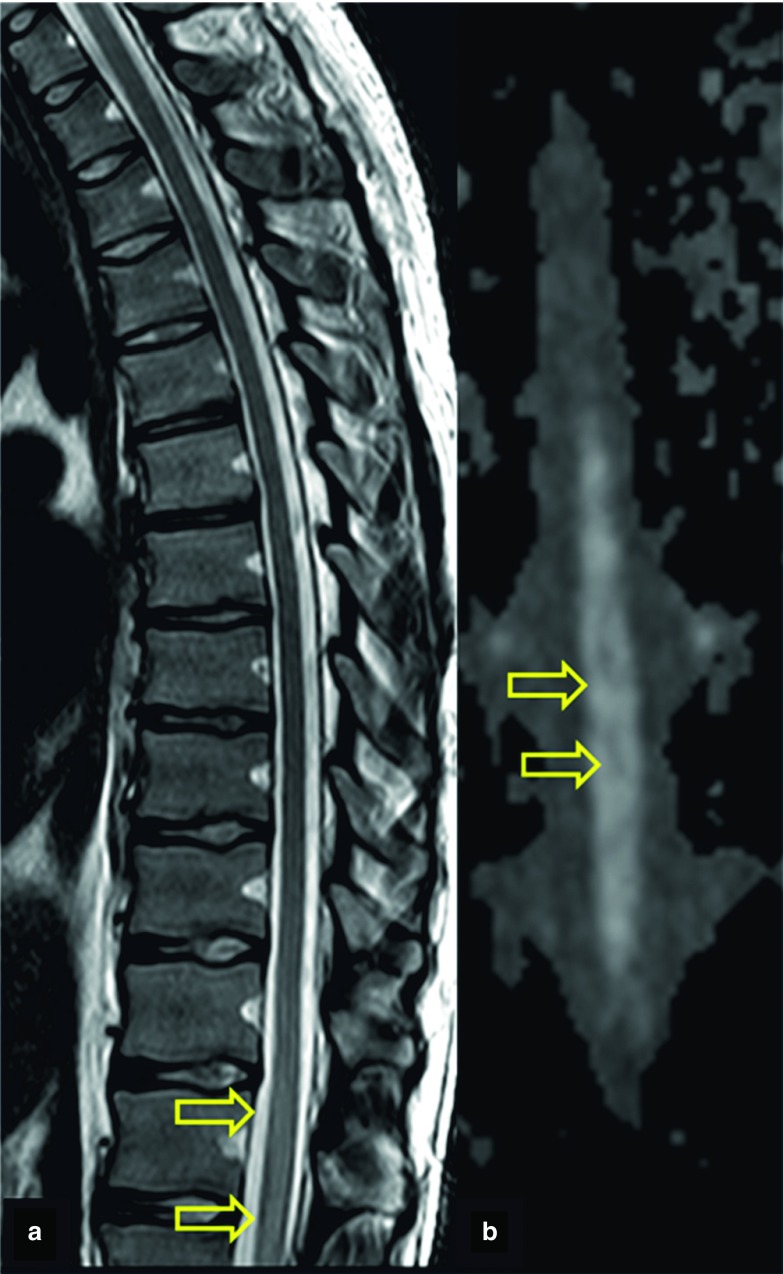
MRI 18 h following onset of symptoms. (a) The sagittal *T*_2_ weighted turbo spin-echo MR image shows oedema and swelling of the conus medullaris (arrows). (b) Low apparent diffusion coefficient is shown in keeping with restricted diffusion, which supports the diagnosis of acute ischaemia (arrows).

The initial MRI of the whole spine, performed approximately 18 h after onset of symptoms, showed high signal intensity on *T*_2_ weighted images, in keeping with diffuse oedema within the medullary conus. In addition, it showed fusiform swelling at the level of T12 along with restricted diffusion (Figure 1). The findings were compatible with an ischaemic insult.

## TREATMENT

Based on imaging and clinical findings, intravenous (i.v.) steroids were administered without any clinical improvement.

## OUTCOME AND FOLLOW-UP

**Figure 2.**
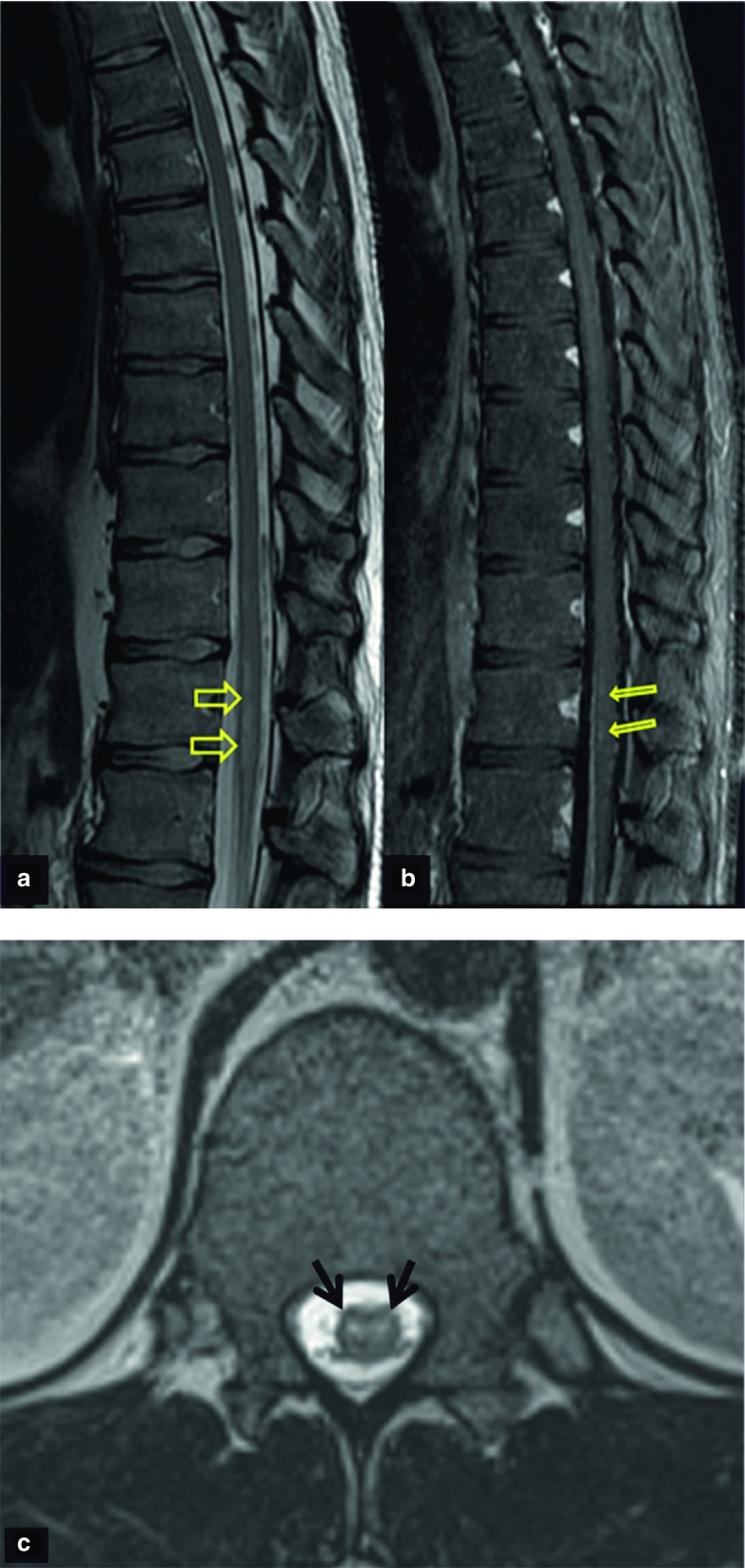
A follow-up MRI 28 days after the initial one shows more precise borders of the lesion on the *T*_2_ weighted sagittal image [arrows in (a)] and foci of enhancement on the fat-suppressed contrast-enhanced *T*_1_ weighted MR image [arrows in (b)] in keeping with subacute ischaemia. The axial three-dimensional *T*_2_ weighted MR image shows swelling and oedema within the cord at the T12 level [arrows in (c)].

**Figure 3.**
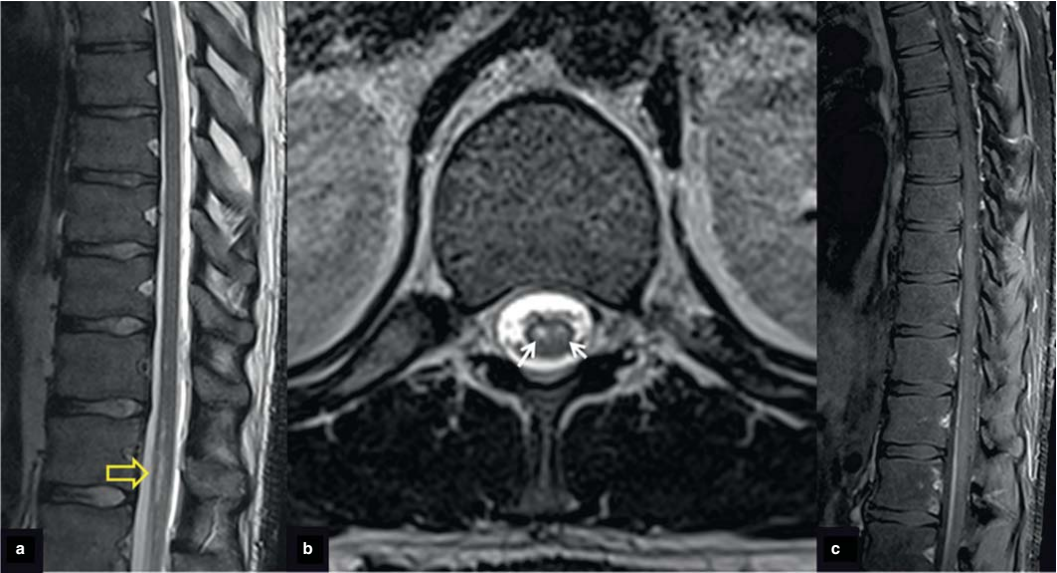
A follow-up MRI 6 weeks after the initial one shows well-defined borders of the lesion on the sagittal (a) and axial (b) *T*_2_ weighted MR images (arrows) and no enhancement on the fat-suppressed contrast-enhanced *T*_1_ weighted MR image (c).

**Figure 4.**
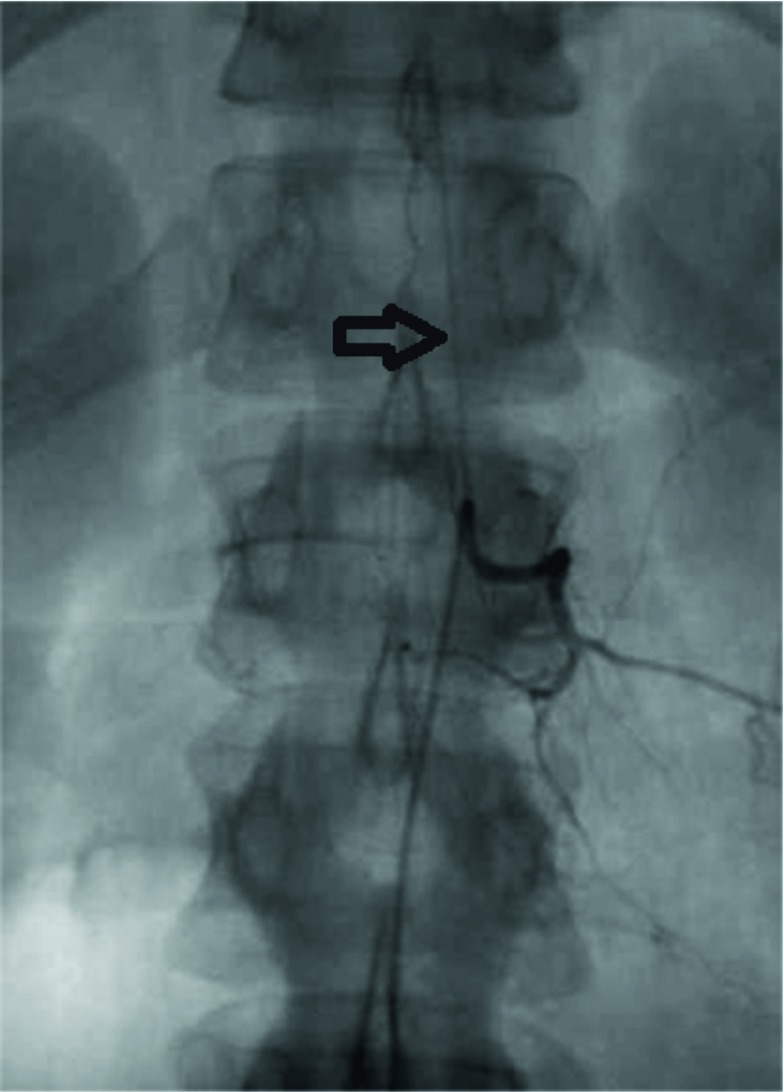
Left lumbar selective arteriography at the level of the L1 vertebral body showing the normal anatomy of an ascending radiculomedullary artery (arrow). All other possible arterial feeding branches were also opacified without evidence of vascular disorders.

Follow-up MRI at 4 weeks showed sharp borders of the high-signal-intensity areas in the conus medullaris and patchy foci of enhancement after i.v. contrast administration (Figure 2). The lesion did not progress to the lower spinal cord. The findings suggest evolution of the lesion to subacute ischaemia. A third MRI examination at 6 weeks revealed no contrast enhancement in keeping with transition of the lesion to a chronic stage (Figure 3). 5 weeks following the onset of symptoms, a detailed selective arteriography of multiple spine levels revealed no impaired vascularization of the anterior spinal artery or the conus medullaris, or any spinal dural arteriovenous fistula (Figure 4).

The patient regained complete bowel control and normal sexual function 2 weeks after the onset of symptoms along with significant reduction of pain. 12 weeks after the onset of symptoms, the lower limb paraesthesia did not show any significant improvement. He was unable to control the bladder function and permanent urinary catheterization was necessary. As a complication, a bladder stone measuring 4.5 cm in diameter was found 8 months later, which required open surgery for removal. The patient was scheduled for an outpatient intense rehabilitation programme. 1 year after symptom onset, he had regained full bladder control. The 28-month clinical follow-up showed a mild waddling gait and the patient was unable to run. Other than this, the complete clinical examination did not reveal any additional pathological findings.

## Discussion

SUP surfing is a rapidly disseminating trend among athletes from all around the world. It is the use of a paddle that makes it easier than ocean surfing. Moreover, there is no need for extreme wave heights to practise SUP surfing, a feature that makes it popular not only across ocean coasts but also in places with lakes, rivers and coasts where mild wind blows. In addition, all the features that make SUP surfing much easier than ocean surfing have guaranteed until now an increased level of safety. Thus, a literature search with keywords “stand up paddle”, “surfing injuries”, “SUP”, yields only one report to date.^1^

Myelopathy, although rare, is an important and alarming complication reported in ocean surfers.^2^ It has been reported to affect athletes even from their very first surfing lesson. Its aetiology is still not clear, as the condition capable of producing such spinal cord lesions has not been identified using imaging techniques. However, several possible pathophysiological mechanisms have been proposed, all resulting in spinal cord ischaemia. They either include mechanisms of blood flow insufficiency of the artery of Adamkiewicz or perforating vessels,^2^ or describe infarction scenarios, such as venous infarction resulting from decreased blood flow through inferior vena cava caused by lumbar hyperextension or fibrocartilaginous embolism triggered by accidental Valsava manoeuvres while standing.^3^ Clinical examination shows overt neurological deficit and MRI reveals a “pencil-like” zone of increased cord *T*_2_ signal extending to a variable cord length, from the upper thoracic levels to the conus medullaris.^3–9^

To the best of our knowledge, SUP surfing has not yet been linked with any disorders, either traumatic or non-traumatic. This is one of the reasons why this sport has become popular even among young children, as it is considered to be easy and safe. Therefore, our report of such an important complication raises concerns regarding the absolute safety of SUP surfing. Our patient (a football teacher) was fit and well trained, but developed myelopathy during his first SUP surfing experience. This underlies the fact that experience and previous training do not play an important role in the possibility of developing myelopathy.

Moreover, detailed history as well as clinical and radiological examination did not reveal any of the factors previously described to predispose our patient to surfer’s myelopathy, such as a thin body habitus, weak musculature, prolonged travel time, hypercoagulation, central canal stenosis or spondylolisthesis.^2,3^ However, SUP surfing may involve prolonged use of the prone position on a board, leading to lumbar spine hyperlordosis, before standing up to use the paddle to propel forward to catch the waves. This occurs in high waves where paddling while standing is difficult or impossible when going in, away from the coast, for further action. Thus, we assume that a possible reason could be transient cord ischaemia, caused by temporary vessel occlusion during the hyperextension periods with or without associated dehydration.

Our differential diagnosis includes multiple sclerosis, acute transverse myelitis, acute disseminating encephalomyelitis, intramedullary neoplasms and arteriovenous fistulae. However, all these conditions were excluded by means of MRI and angiography. Moreover, the clinical course of our patient, as well as the acute presentation of myelopathy, is not in line with such disorders.

Interestingly, at the 2-year follow-up, our patient had not regained full functionality. This is an important fact, bearing in mind that such a complication would affect the rest of an athlete’s life. Thus, attention should be drawn towards proper education and practice by young people wishing to be involved in SUP surfing.

In conclusion, myelopathy may develop in SUP surfers. It is a serious disorder that can appear soon after the first SUP practice session and is probably caused by transient spinal cord ischaemia resulting from lumbar hyperlordosis while lying prone on the surfboard.

## LEARNING POINTS

1. SUP surfing is rarely associated with surfer’s myelopathy.
2. MRI in the early phase after the onset of symptoms is the method of choice for depicting abnormal findings in the spinal cord.
3. The selective arteriography is usually normal and thus a diagnosis of congenital dural malformation can be excluded.
